# Reactivation of Human X-Linked Gene and Stable X-Chromosome Inactivation Observed in Generation and Differentiation of iPSCs from a Female Patient with HNRNPH2 Mutation

**DOI:** 10.3390/cells14191486

**Published:** 2025-09-23

**Authors:** Guibin Chen, Alexander Rodriguez-Lopez, Darawalee Wangsa, Richa Madan Lomash, Xiuli Huang, Catherine Z. Chen, Rodney A. Bowling, Neda Ghousifam, Courtney J. Banks, Kerstin A. Hurd, Jizhong Zou, Wei Zheng

**Affiliations:** 1 National Center for Advancing Translational Sciences, National Institutes of Health, 9800 Medical Center Drive, Rockville, MD 20892, USA; guibin.chen@nih.gov (G.C.); alexander.rodriguezlopez@nih.gov (A.R.-L.); darawalee.zong@nih.gov (D.W.); richa.lomash@nih.gov (R.M.L.); xiuli.huang@nih.gov (X.H.); catherine.chen@nih.gov (C.Z.C.); 2 To Cure a Rose Foundation, Building 4000, 6101 Highland Campus Dr #2250, Austin, TX 78752, USA; rodney@tocurearose.org (R.A.B.J.); neda@tocurearose.org (N.G.); courtney@tocurearose.org (C.J.B.); 3 National Heart, Lung, and Blood Institute, National Institutes of Health, 10 Center Drive, Bethesda, MD 20892, USA; kerstin.hurd@nih.gov

**Keywords:** X chromosome inactivation, Bain type X-linked intellectual disability syndrome, HNRNPH2, iPSC

## Abstract

X chromosome inactivation (XCI) is a fundamental epigenetic process that balances X-linked gene expression between females and males by silencing one X chromosome in female cells. Variability or skewing of XCI can influence the clinical presentation of X-linked disorders. Bain type X-linked intellectual disability syndrome (MRXSB), caused by mutations in the X-linked *HNRNPH2* gene, is characterized by intellectual disability, developmental delay, and neurological abnormalities. In female patients, XCI heterogeneity complicates disease modeling and therapeutic development. Induced pluripotent stem cells (iPSCs) offer a unique platform to study patient-specific disease mechanisms, but the dynamics of XCI during iPSC reprogramming, maintenance, and differentiation are not fully understood. In this study, we generated 12 iPSC clones from fibroblasts of a female MRXSB patient heterozygous for the *HNRNPH2* c.340C > T mutation. Four clones expressed the mutant *HNRNPH2* allele and eight expressed the wild-type allele, indicating X chromosome reactivation (XCR) followed by random XCI during reprogramming. Importantly, these XCI patterns remained stable during long-term iPSC propagation and subsequent differentiation into the three germ layers and neural stem cells. Our findings provide new insights into XCI and XCR dynamics in the context of X-linked neurodevelopmental disorders and emphasize the importance of careful clone selection for accurate disease modeling using iPSC-based approaches.

## 1. Introduction

Bain type X-linked intellectual disability syndrome (MRXSB) is a neurodevelopmental disorder caused predominantly by de novo, but occasionally inherited, mutations in the *HNRNPH2* gene on the X chromosome [1,2,3,4,5,6,7]. Both male and female patients are affected, presenting with intellectual disability, developmental delay, and neurological abnormalities. *HNRNPH2* encodes a 449–amino acid RNA-binding protein containing three RNA recognition motifs (RRMs) and two glycine-rich domains (GRDs). The nuclear localization sequence (NLS) within the GRDs (amino acids 194–220) is critical for the protein’s function in RNA transport, and most pathogenic *HNRNPH2* mutations cluster in this region [8]. Despite its recognized role in brain development and neurological function, the precise molecular mechanisms underlying MRXSB remain largely unclear, and no effective treatments currently exist.

In female cells, one of the two X chromosomes undergoes X-chromosome inactivation (XCI), an epigenetic process ensuring dosage compensation of X-linked genes between males and females. However, XCI can be incomplete or skewed, leading to variable patterns of gene expression that may influence disease severity in X-linked disorders. For example, skewed XCI can mitigate or exacerbate the clinical phenotype, and in some cases, women with pathogenic *HNRNPH2* variants may remain asymptomatic due to complete inactivation of the mutant allele [3]. Understanding the dynamics of XCI is therefore essential for elucidating disease pathophysiology and developing accurate disease models.

Induced pluripotent stem cells (iPSCs) have revolutionized the modeling of human genetic diseases, enabling the generation of patient-specific cell lines that can differentiate into diverse cell types. iPSCs are invaluable for studying disease mechanisms, screening therapeutics, and investigating developmental biology. However, the process of reprogramming somatic cells into iPSCs can alter the X chromosome’s epigenetic state [9]. While this phenomenon enables the generation of isogenic female iPSC lines that express either the wild-type or mutant allele of X-linked genes, it also introduces uncertainty regarding the status and stability of XCI during iPSCs derivation, propagation, and differentiation. Human iPSCs can display a spectrum of XCI states—including active (Xa), inactive (Xi), or partially reactivated (Xe) X chromosomes—depending on factors such as the reprogramming method and cellular environment [10,11]. This variability can affect both differentiation potential and the utility of iPSCs in disease modeling.

In this study, we investigated the dynamics and stability of XCI during iPSCs generation, propagation, and differentiation using fibroblasts from a female MRXSB patient carrying a heterozygous c.340C > T mutation in HNRNPH2. We generated iPSC clones expressing either the mutant or wild-type HNRNPH2 allele, reflecting X chromosome reactivation (XCR) and subsequent XCI during reprogramming. We further examined the maintenance of XCI patterns during extended iPSCs culture and differentiation into the three germ layers and neural stem cells. Our findings highlight the importance of careful allele-specific characterization and selection of iPSC clones for accurate modeling of X-linked disorders and provide new insights into XCI dynamics relevant for disease modeling and therapeutic development in MRXSB.

## 2. Materials and Methods

### 2.1. Cell Culture

Primary skin fibroblasts were obtained from a female patient carrying the *HNRNPH2* R114W (c.340C > T) mutation. A 3 mm punch biopsy was enzymatically dissociated, and single-cell-derived fibroblast clones were expanded in 96-well plates. Allele- discriminating TaqMan™ real-time PCR was used to identify clones expressing the mutant or wild-type (WT) *HNRNPH2* allele, yielding approximately a 48:52 ratio of R114W-positive to WT-positive clones. A fibroblast line expressing the *HNRNPH2*-R114W allele was selected for reprogramming. Fibroblasts were cultured in DMEM supplemented with 10% fetal bovine serum (FBS) and maintained at 37 °C, 5% CO_2_. Induced pluripotent stem cells (iPSCs) were seeded on Matrigel (Corning, Austin, TX, USA, 354277)-coated plates and maintained in StemFlex medium (Gibco, Waltham, MA, USA, A33493-01) at 37 °C with 5% CO_2_ and 5% O_2_. Cells were passaged using EZ-LiFT™ (Sigma-Aldrich, Saint Louis, MO, USA) upon reaching 70–80% confluency.

### 2.2. Reprogramming of Human Skin Fibroblasts

Fibroblasts were reprogrammed into iPSCs using Sendai virus vectors encoding OCT4, KLF4, SOX2, and C-MYC (A16517, Thermo Fisher, Waltham, MA, USA), following established protocols [12]. The resulting p0 iPSCs pool was single-cell sorted into 96-well plates to derive clonal iPSC lines.

### 2.3. Monolayer Differentiation Assay

The differentiation potential of iPSC lines was assessed at passage 15 using the STEMdiff™ Trilineage Differentiation Kit (STEMCELL Technologies, Vancouver, BC, Canada), following the manufacturer’s instructions. Briefly, iPSCs were plated onto Matrigel-coated plates in StemFlex medium overnight, then switched to lineage-specific STEMdiff™ Trilineage media for ectoderm, mesoderm, or endoderm differentiation, with medium changes every other day. Cells were harvested for analysis of lineage-specific markers at day 5 (mesoderm, endoderm) or day 7 (ectoderm).

### 2.4. Neural Stem Cell (iNSC) Differentiation and Expansion

iPSC lines were differentiated into neural stem cells (iNSCs) using the PSC Neural Induction Medium kit (Gibco), containing Neurobasal^®^ Medium and Neural Induction Supplement. iPSCs were seeded onto Matrigel-coated 6-well plates at a density of 2.5 × 10^5^–3 × 10^5^ cells per well. After 24 h, the medium was replaced with neural induction medium, with changes every other day. By day 7, a homogeneous population of iNSCs was obtained and subsequently expanded in Knockout DMEM/F12 supplemented with StemPro™ Neural Supplement (Gibco), 20 ng/mL bFGF, 20 ng/mL EGF, and 50 μg/mL PenStrep (Gibco). Cells were passed as needed for up to 20 passages.

### 2.5. Gene Expression Analysis by RT-qPCR

Total RNA was extracted from iPSCs and differentiated cells using the RNeasy Mini Kit (Qiagen, Venlo, The Netherlands). cDNA was synthesized using SuperScript™ III (Invitrogen, Carlsbad, CA, USA) according to the manufacturer’s protocol. Gene expression was assessed by quantitative real-time PCR (RT-qPCR) using TaqMan™ probes (Thermo Fisher Scientific, Waltham, MA, USA) for pluripotency (OCT4, NANOG), mesoderm (T, HAND1), endoderm (SOX17, FOXA2), and ectoderm (SOX1, PAX6) markers. All reactions were performed in triplicate using 2X Advanced TaqMan™ PCR Master Mix on a ViiA™ 7 Real-Time PCR System (Thermo Fisher Scientific). Expression levels were normalized to the housekeeping gene RPLP0.

### 2.6. DNA Sequencing

Genomic DNA was extracted from iPSC clones using the QIAamp™ DNA Mini Kit (Qiagen). PCR amplification was performed with Q5™ High-Fidelity Master Mix (NEB) using a 2720 Thermal Cycler (Applied Biosystems, Waltham, MA, USA). The PCR program consisted of initial denaturation at 98 °C for 30 s, followed by 35 cycles of 98 °C for 10 s, 60 °C for 15 s, and 72 °C for 30 s, with a final extension at 72 °C for 5 min. PCR products were sequenced by Eurofins Genomics, and sequence analysis was performed using ApE v.0.55 and SnapGene v. 8.0.2 software. Primer sequences are provided in Supplementary Table S1.

### 2.7. Immunocytochemistry

Cells were seeded on Matrigel-coated 96-well plates and fixed with 4% paraformaldehyde for 10 min at room temperature. After PBS washes, cells were permeabilized with 0.3% saponin in PBS for 15 min and blocked for 30 min with Cell Staining Buffer (BioLegend, San Diego, CA, USA). Primary antibodies against SOX2, OCT4, SSEA4, NANOG, TRA-1-60, SOX1, PAX6, and Nestin were applied in blocking buffer and incubated overnight at 4 °C. After washing, appropriate secondary antibodies (see Supplementary Table S2) were incubated for 1 h at room temperature. Nuclei were counterstained with Hoechst 33342 for 15 min. Images were captured using an ImageXpress Confocal HT.ai system (Molecular Devices, San Jose, CA, USA) with a 10X objective. Scale bar: 400 µm.

### 2.8. Flow Cytometry Analysis

iPSCs at passage 10 were dissociated with TrypLE (Thermo Fisher), fixed with 4% paraformaldehyde, and permeabilized with 0.3% saponin. Cells were stained with fluorophore-conjugated antibodies (see Supplementary Table S2) for 30 min at room temperature. Data acquisition was performed on a NovoCyte Quanteon flow cytometer (Agilent, Santa Clara, CA, USA), and analysis was conducted using appropriate software.

## 3. Results

### 3.1. X Chromosome Reactivation and Inactivation in Human iPSC Clones Generated from Female MRXSB Patient Fibroblasts

**Figure 1:**
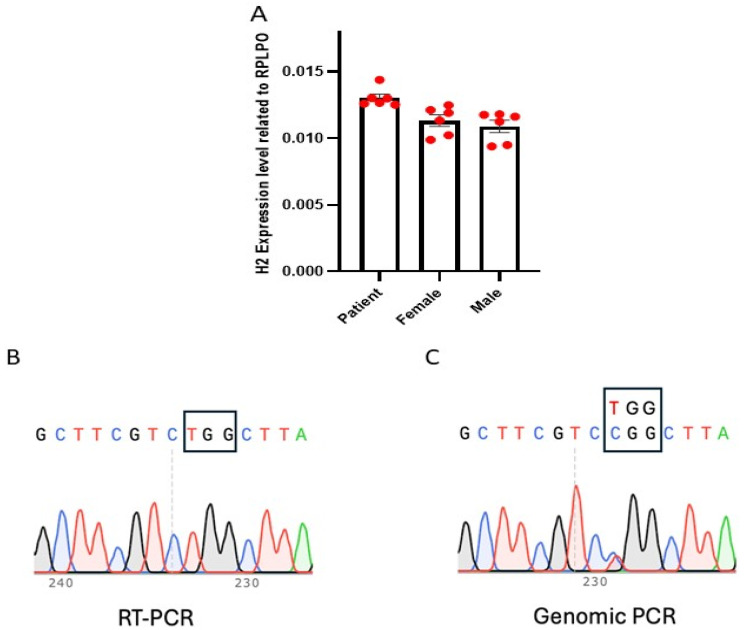
Expression of *HNRNPH2* gene in female patient fibroblast. (**A**) Expression level of *HNRHPH2* in patient fibroblast (Patient)is comparable to that of wild-type control male (Male) and female fibroblast (Female). The red dots indicate technical replicates, and the error bars represent the standard deviation (SD). (**B**) Sanger sequencing of RT-PCR product from patient fibroblast only showed expression of mutant allele (TGG in the box). (**C**) Sanger sequencing of genomic PCR products from patient fibroblast showed existence of both wild-type (CGG in the box) and mutant allele (TGG in the box) with about 1:1 ratio.

We obtained fibroblasts from a female MRXSB patient carrying the *HNRNPH2* R114W (CGG > TGG) mutation (c.340C > T in the gene) and established a single-cell-derived clonal fibroblast line expressing *HNRNPH2* R114W. This mutation affects a highly conserved arginine residue within the RNA-recognition motif (RRM), which is critical for RNA processing and splicing regulation. When compared to age-matched wild-type female and male fibroblasts, the expression level of *HNRNPH2* in the patient’s fibroblasts was found to be comparable, as assessed by real-time RT-PCR (Figure 1A). To evaluate X-chromosome inactivation in the fibroblast cells, we designed specific primers targeting the mutated locus containing c.340C > T in the *HNRNPH2* gene. Following RT-PCR, sequencing results revealed the presence of c.340C > T mutation in the RT-PCR product indicated expression of an active mutant allele (Xa^Mu^Xi^WT^). These findings indicate that the wild-type allele of the X chromosome was inactivated (Figure 1B) while the mutant allele remained active in the patient fibroblast. We also used genomic PCR to confirm that the patient fibroblast is heterozygous for HNRNPH2 R114W mutation (Figure 1C). Given the absence of this mutation in the parents and a 1:1 wild-type to mutant allele ratio in the patient’s fibroblasts, the mutation was determined to be de novo, likely arising during early embryonic development or due to germline mosaicism.

**Figure 2:**
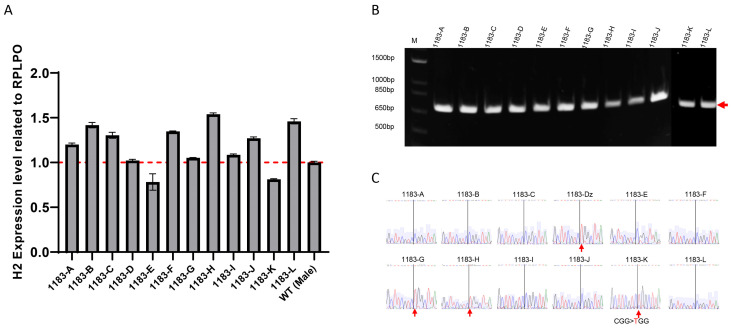
Reactivation of Human X-Linked Gene During iPSCs Generation from Fibroblasts. We generated 12 human induced pluripotent stem cells (iPSCs) clones from the fibroblasts of a female patient carrying a heterozygous mutation in the *HNRNPH2* gene. RT-PCR analysis showed that the expression level of *HNRNPH2* is similar to that of the iPSCs derived from a healthy male volunteer (**A**). The result indicated that one of the two X-chromosomes is inactive in these cells. Sanger sequencing (**B**) of RT-PCR products (**C**) confirmed that XCI of the mutant allele was observed in 8 out of 12 clones while 4 out of 12 clones retained XCI of the wild-type allele. Red arrows in (**B**) indicate RT-PCR product and in (**C**) indicate CGG > TGG mutation.

Fibroblasts were reprogrammed into iPSCs using the integration-free CytoTune-Sendai viral vector system using established protocol [12] which delivers the transcription factors OCT4, KLF4, SOX2, and C-MYC. On day 21 of reprogramming, we selected 12 clones exhibiting typical embryonic stem cells or iPSCs morphology for further expansion and characterization. At passage 8, cells were collected to assess *HNRNPH2* expression levels by RT-PCR. The results indicated that *HNRNPH2* expression in these cells was comparable to iPSCs derived from a healthy male volunteer (Figure 2A), despite variations among clones. Sequencing of RT-PCR products from individual iPSC clones showed allele-specific expression of mutated allele in 4 out of 12 clones and expression of wild-type allele in 8 out of 12 clones (Figure 2B,C). We did not observe any colony expressing both WT and mutant *HNRNPH2* gene. Because the patient fibroblast only has active mutant allele (Xa^Mu^Xi^WT^) and iPSC clones have either active wild-type allele (Xa^WT^Xi^Mu^) or mutant allele (Xa^Mu^Xi^WT^). The findings suggested X-chromosome reactivation (XCR) of wild-type allele occurred in the early phase of reprogramming, resulting in Xa^WT^Xa^Mu^, followed by clonal XCI in the late phase of reprogramming.

### 3.2. Allele-Specific Expression Analysis Demonstrates Stable XCI During iPSCs Propagation

**Figure 3:**
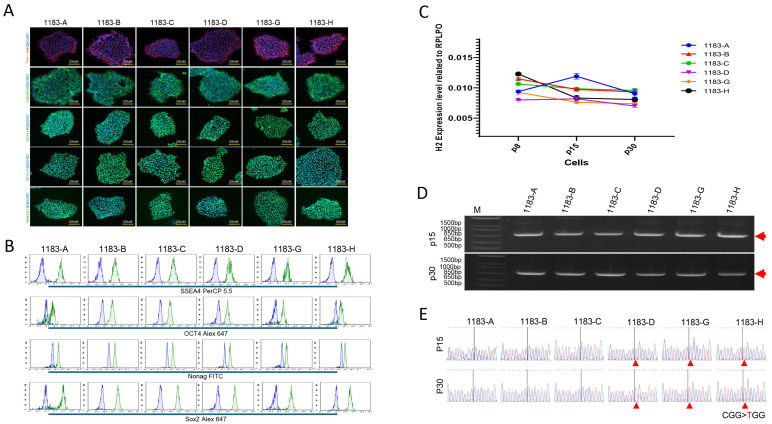
Allele-Specific Expression Analysis Demonstrates Stable XCI During iPSCs Propagation. Six iPSC clones were selected and expanded for a comprehensive analysis of genotype, phenotype, and pluripotent potential. All iPSC clones exhibited typical morphology and expressed the pluripotency markers NANOG, SOX2, OCT4, SSEA4, and TRA-1-60, but not neural differentiation marker NESTIN, as confirmed by immunofluorescence staining and flow cytometry (**A**,**B**). We analyzed HNRNPH2 expression during iPSCs propagation at passages 8, 15, and 30 using RT-PCR. The results indicated that HNRNPH2 expression remained relatively stable over time (**C**). Furthermore, we demonstrated that once established, X-chromosome inactivation (XCI) remained unchanged throughout iPSCs expansion (**D**,**E**). Red arrows in (**D**) indicate RT-PCR product and in (**C**) indicate CGG > TGG mutation.

The status of the X chromosomes is apparently extremely sensitive to culturing conditions and the factors that allow human ES cells with two active X chromosomes to be reliably propagated remains uncertainty. To evaluate XCI status during iPSCs propagation, six iPSC clones, including three Xa^WT^Xi^Mu^ and three Xa^Mu^Xi^WT^, were selected and expanded for a comprehensive analysis of genotype, phenotype, pluripotent potential, and XCI stability. All 6 iPSC clones have heterozygous R114W mutation in *HNRNPH2* gene (Figure S1) that exhibited typical morphology of iPSCs (Figure S2) and expressed the pluripotency markers of NANOG, SOX2, OCT4, SSEA4, and TRA-1-60, confirmed by immunofluorescence staining and flow cytometry (Figure 3A,B). We continuously cultured and expanded the iPSC clones that were analyzed for *HNRNPH2* expression during iPSCs propagation at passages 8, 15, and 30 using real-time RT-PCR. The results indicated that *HNRNPH2* expression remained consistent during expansion (Figure 3C). Furthermore, we followed up the states of XCI in the iPSCs propagation and found that each clone retained its respective XCI status—either wild-type (Xa^WT^Xi^Mu^) or mutant allele expression (Xa^Mu^Xi^WT^)—across all passages (Figure 3D,E). These results demonstrate that once established, XCI is stable during long-term iPSCs culture.

### 3.3. The Three Germ Layer Cells Derived from iPSCs Exhibited the Same XCI Pattern as Their Parental Cells

**Figure 4:**
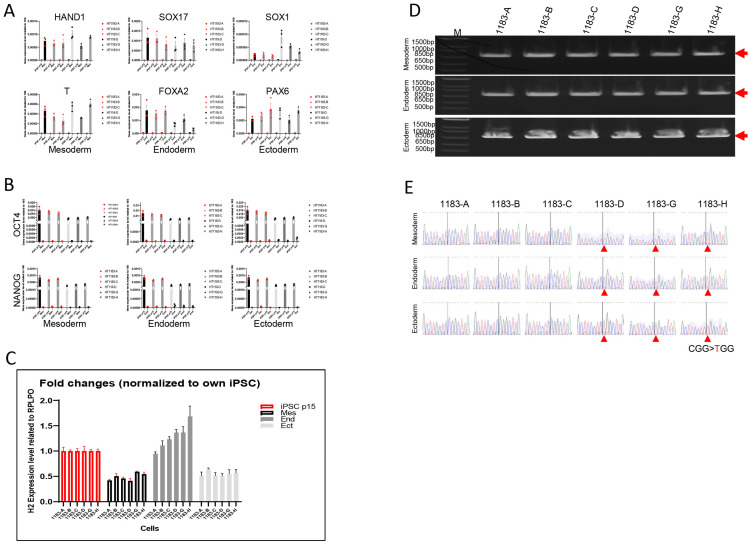
The three germ layer cells derived from iPSCs exhibited the same XCI pattern as their parental cells. To validate their differentiation potential, a monolayer differentiation assay was performed, demonstrating the expression of representative genes: SOX17 and FOXA2 (endoderm), HAND1 and T (mesoderm), and SOX1 and PAX6 (ectoderm), as assessed by RT-PCR (**A**). Additionally, the loss of endogenous pluripotency gene expression (OCT3/4 and NANOG) was confirmed (**B**). These results indicate that all iPSC lines retained the ability to differentiate into the three germ lineages in vitro. Compared to iPSCs, HNRNPH2 expression was downregulated in both mesoderm and ectoderm lineages, while slightly upregulated in the endoderm lineage (**C**). Allele-specific expression analysis further confirmed the stable inactivation of one of the two X chromosomes in iPSC-derived three germ layer cells, maintaining the same XCI pattern in their parental cells (**D**,**E**). Red arrows in (**D**) indicate RT-PCR product and in (**E**) indicate CGG > TGG mutation.

To access both the differentiation potential and XCI stability of these iPSCs, we performed a monolayer differentiation assay to direct iPSCs into the three germ layer lineages. Morphological changes during differentiation were monitored (Supplementary Figure S2). We then analyzed the gene expression profiles of the differentiating cells, which demonstrated upregulation of representative lineage-specific genes: *SOX17* and *FOXA2* (endoderm) [13], *HAND1* and *T* (mesoderm), and *SOX1* and *PAX6* (ectoderm) [14], as assessed by RT-PCR (Figure 4A). Additionally, the loss of endogenous pluripotency gene expression (*OCT3/4* and *NANOG*) was confirmed across all three germ layer lineages (Figure 4B), indicating successful differentiation. Compared to the stage of iPSCs, *HNRNPH2* expression was downregulated in mesoderm and ectoderm lineages but slightly upregulated in the endoderm lineage (Figure 4C). Allele-specific expression analysis further confirmed stable expression of either mutant or wild-type *HNRNPH2* allele in iPSC-derived germ layer cells, maintaining the same XCI pattern as in their parental iPSCs (Figure 4D,E).

### 3.4. The Neural Stem Cells Derived from iPSCs Maintain Stable XCI Patterns

**Figure 5:**
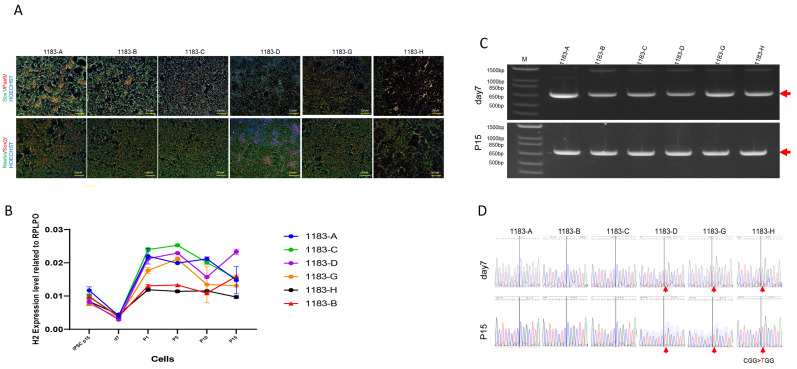
The neural stem cells generated from iPSC lines maintained a stable XCI pattern. The iPSC clones were differentiated into neural stem cells (iNSCs), characterized by the expression of Nestin, SOX1, SOX2, and PAX6 (**A**). The expression level of *HNRNPH2* was downregulated when iPSCs were induced into iNSCs at day 7. However, *HNRNPH2* expression remained relatively stable during iNSC expansion until passage 15 (**B**). RT-PCR and sequencing analyses further confirmed that neural induction and iNSCs maintained the XCI status observed in their parental iPSC lines (**C**,**D**). Red arrows in (**C**) indicate RT-PCR product and in (**D**) indicate CGG > TGG mutation.

MRXSB is a neurodevelopmental disorder caused by mutations in the *HNRNPH2* gene, leading to intellectual disability and cognitive impairments. Neural stem cells (NSCs) with stable allele-specific expression of *HNRNPH2* from MRXSB patient iPSCs are a useful tool to study underlying mechanisms of the disease and to develop assays for therapeutics development [15]. Six iPSC clones (three Xa^WT^Xi^Mu^ and three Xa^Mu^Xi^WT^) derived from patient fibroblasts with the *HNRNPH2* mutation were differentiated into induced neural stem cells (iNSCs), which were characterized by the expression of neural stem cell markers NESTIN, SOX1, SOX2, and PAX6 (Figure 5A). The expression level of *HNRNPH2* was downregulated at day 7 during iPSCs differentiation into iNSCs. To further investigate *HNRNPH2* expression dynamics, we tracked its levels at various time points during iNSC expansion (Figure 5B). The experiments with RT-PCR and sequencing analyses confirmed that the *HNRNPH2* expression remained relatively stable in the same XCI status during neural induction from iPSCs to iNSCs and iNSC passaging up to passage 15, similar to their parental iPSC lines (Figure 5C,D).

## 4. Discussion

In this study, we successfully generated iPSC lines from fibroblasts of a female MRXSB patient carrying a heterozygous mutation in the *HNRNPH2* gene located on the X-chromosome using the integration-free CytoTune-Sendai viral vector system. Our findings provide significant insights into the stability of X-chromosome inactivation (XCI) during iPSCs reprogramming, propagation, and differentiation, as well as the expression dynamics of *HNRNPH2* in pluripotent and lineage-specific cells.

A major observation in our study was the reactivation of the silenced X-linked *HNRNPH2* gene during the reprogramming process. It was not clear that during reprogramming to iPSCs whether the inactive X chromosome can be reactivated, resulting in a biallelic expression of X-linked genes, similar to the state in early embryogenesis. Although some previous reports have suggested that XCI can be maintained during reprogramming [16,17], our results support the model in which X-chromosome reactivation (XCR) can occur, followed by a new round of random XCI during iPSCs establishment. Specifically, we generated 12 iPSC clones from patient fibroblasts that originally expressed only the mutant HNRNPH2 allele. Upon characterization, eight clones reactivated the previous inactivated Xi^WT^ allele and switched to Xa^WT^Xi^Mu^ while four clones retained Xa^Mu^ allele (Xa^WT^Xi^Mu^), indicating that XCR and subsequent stochastic XCI occurred during reprogramming. This agrees with previous findings that reprogramming can induce XCR and allow for the derivation of isogenic iPSC lines expressing either allele from the same patient sample [9]. Kim et al. conducted a study using heterozygous female fibroblast lines that only express the mutant HPRT allele. Interestedly, they observed the expression of the wild-type HPRT allele both during the reprogramming phase and in the fully reprogrammed iPSC clones [9]. One of the hypotheses is that the XCR observed in our reprogramming experiment was due to high-efficiency reprogramming or high-level expression of reprogramming factors by Sendai Virus, similar to high copy number of reprogramming virus observed by Kim et al. [9] Another hypothesis is that wild-type *HNRNPH2* expressing (Xa^WT^Xi^Mu^) iPSCs may have survival advantage over mutant *HNRNPH2* expressing (Xa^Mu^Xi^WT^) iPSCs during reprogramming, as we isolated many more Xa^WT^Xi^Mu^ clones than Xa^Mu^Xi^WT^ clones. The benefit of XCR followed by XCI during reprogramming is that it allowed us to generate normal isogenic control clones (Xa^WT^Xi^Mu^) and mutant clones (Xa^Mu^Xi^WT^) from the same patient sample in one reprogramming experiment. The Xa^WT^Xi^Mu^ iPSCs are also stable after extensive expansion and differentiation, providing unlimited cell resource for drug screening. Additionally, the overall expression level of *HNRNPH2* in patient-derived iPSCs was comparable to that of male control iPSCs, suggesting that XCI status plays a key role in regulating gene dosage in these cells.

We further analyzed the stability of XCI and *HNRNPH2* expression during iPSCs propagation over multiple passages. The results demonstrated that once XCI was established in a given iPSC line (expressing either mutant or wild-type allele), the XCI pattern remained stable through extended culture (up to passage 30). This finding is crucial, as XCI instability in female iPSCs has been reported [17]. The ability to maintain a stable XCI pattern ensures that these iPSC lines can serve as reliable models for further studies.

Importantly, the iPSC lines expressing either wild-type or mutant *HNRNPH2* demonstrated robust differentiation potential into the three germ layers, as confirmed by the expression of lineage-specific markers. Interestingly, allele-specific expression analysis showed that the XCI pattern observed in parental iPSCs was preserved in differentiated derivative cells such as neural stem cells and neurons. This indicates that XCI remains consistent during differentiation, supporting the reliability of these cells for disease modeling. Notably, *HNRNPH2* expression was downregulated in mesodermal and ectodermal derivatives but slightly upregulated in endodermal cells. Notably, *HNRNPH2* expression was downregulated in mesodermal and ectodermal derivatives but slightly upregulated in endodermal cells. This differential expression of *HNRNPH2* across germ layers may reflect its involvement in regulatory pathways critical for lineage specification. Several studies have highlighted the importance of lineage-specific gene regulation in development and differentiation. For example, the regulation of X chromosome inactivation can vary between cell types and developmental stages [18,19]. Additionally, lineage-specific transcription factors and epigenetic modifications play crucial roles in driving differential gene expression during development [20,21]. Understanding these mechanisms of lineage-specific regulatory processes governing *HNRNPH2* expression could provide valuable insights into both developmental biology and diseases linked to aberrant X chromosome regulation. To further explore this possibility, future studies investigating *HNRNPH2* expression in primary cell cultures derived from adult tissues of ectodermal, mesodermal, and endodermal lineages would be instrumental in validating our observations and elucidating the underlying regulatory mechanisms.

Generating neural stem cells (NSCs) iPSCs derived from patients with MRXSB is crucial for understanding the disease’s underlying mechanisms and developing potential therapeutic strategies. In the patient-iPSC derived neural stem cells (iNSCs), *HNRNPH2* expression changed during iNSC expansion, while the XCI pattern remained stable and consistent with their parental iPSCs. This indicates that neural stem differentiation does not trigger XCI erosion. This is particularly relevant for modeling neurological diseases associated with HNRNPH2 mutations, as maintaining a consistent XCI profile is critical for faithfully recapitulating disease phenotypes in iPSC derived cells. Therefore, this disease model system provides a valuable platform for studying disease pathophysiology and for assay development for drug screening. Additionally, this platform could be used for personalized medicine development, where switching *HNRNPH2* gene expression status from Xa^Mu^Xi^WT^ to Xa^WT^Xi^Mu^ may help develop potential treatments to restore normal neuronal function.

While our study provides valuable insights into the active/inactive status of the X chromosome through the analysis of HNRNPH2 expression, it is important to acknowledge the limitations inherent in relying on a single X-linked gene. The analysis of HNRNPH2 alone may not fully capture the complexities of XCI during the iPSC generation process. To gain a more comprehensive understanding, future studies should incorporate additional hallmarks of the inactive X chromosome such as H3K27me3 and XIST. Additionally, analysis of other X-linked genes could help confirm whether monoallelic expression observed in HNRNPH2 is representative of broader expression patterns across the X chromosome.

## 5. Conclusions

Our study highlights the successful generation of both phenotypically normal iPSC clones and phenotypically mutant clones from the same fibroblast sample of a female MRXSB patient carrying the *HNRNPH2* mutation. We show that X-chromosome reactivation occurs during the reprogramming process, enabling the derivation of iPSC clones expressing either the mutant or wild-type *HNRNPH2* allele. Importantly, the XCI status established in individual iPSC clones is stably maintained during long-term propagation and throughout directed differentiation into all three germ layers, including neural stem cells. Our results provide valuable insights into XCR and XCI stability during reprogramming, expansion, and differentiation. These findings underscore the necessity of careful allele-specific characterization and selection of iPSC lines for accurate disease modeling of X-linked disorders. Furthermore, the stable maintenance of XCI patterns in differentiated cell types highlights the reliability of these iPSC-based models for investigating the pathophysiology of HNRNPH2-related neurodevelopmental disorders and for supporting therapeutic discovery. This approach provides a robust platform for studying the molecular mechanisms underlying MRXSB and offers new opportunities for the development of personalized treatment strategies.

## Data Availability

Data supporting this study are available from the corresponding author on request.

## Supplementary Materials

The following supporting information can be downloaded at: https://www.mdpi.com/article/10.3390/cells14191486/s1, Figure S1: Sanger sequencing of genomic PCR product from 6 MRXSB iPSC clones showed existence of both wild-type (CGG) and mutant allele (TGG) indicated by arrows; Figure S2: The differentiation of iPSC into the three germ layers (ectoderm, mesoderm, and endoderm) shown distinct morphological changes observed under a microscope. (A). Mesoderm differentiation. (B). Endoderm differentiation. (C). Ectoderm differentiation; Table S1: Primers used for RT-qPCR and PCR; Table S2: Antibodies used for immunocytochemistry and FACS.
