# Supplementary material for: Reactivation of Human X-Linked Gene and Stable X-Chromosome Inactivation Observed in Generation and Differentiation of iPSCs from a Female Patient with HNRNPH2 Mutation
Source: Cells. 2025 Sep 23;14(19):1486. doi: 10.3390/cells14191486 (PMC12523473; doi:10.3390/cells14191486)
Supplement: Supplementary file 1 [file cells-14-01486-s001.zip › cells-3820013-supplementary.pdf]

Table S1. Primers used for RT-qPCR and PCR.

Target	Forward/reverse primer (5′-3′)
HNRNPH2	AGCCGTTTGAGGGAAGAAGG / TCGAGGGGGATCATAGTGGG
HNRNPH2	ACAGACGTCTTACAGAAAAGCTG / TCGAGGGGGATCATAGTGGG

Table S2. Antibodies used for immunocytochemistry and FACS.

	Antibody	Dilution	Company	Cat#	RRID
Primary antibodies	Mouse anti-SSEA4	1:100	MilliporeSigma	MAB4304	AB_177629
	Mouse anti-TRA-1-60	1:150	MilliporeSigma	MAB4360	AB_2119183
	Alexa Fluor 488 anti-SSEA4 Antibody	1:50	BioLegend	330412	AB_1089198
	PE anti-TRA-1-60 Antibody	1:50	BioLegend	330610	AB_2119065
	Alexa Fluor 488 anti-SOX2 Antibody	1:50	BioLegend	656110	AB_2563957
	Alexa Fluor 488 anti-OCT4 Antibody	1:50	BioLegend	653708	AB_2563184
	Alexa Fluor 647 anti-NANOG Antibody	1:50	BioLegend	674210	AB_2650619
Secondary antibodies	Alexa Fluor 594 Donkey anti-rabbit	1:300	Life Technologies	A21207	AB_141637
	Alexa Fluor 594 Donkey anti-mouse	1:300	Life Technologies	A21203	AB_141633
	Alexa Fluor 488 Donkey anti-mouse	1:300	Life Technologies	A21202	AB_141607

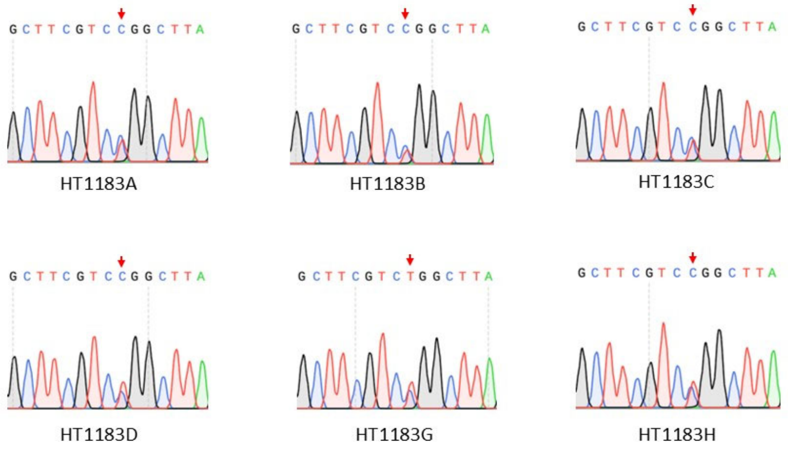

Figure S1. Sanger sequencing of genomic PCR product from 6 MRXSB iPSC clones showed existence of both wild-type (CGG) and mutant allele (TGG) indicated by arrows.

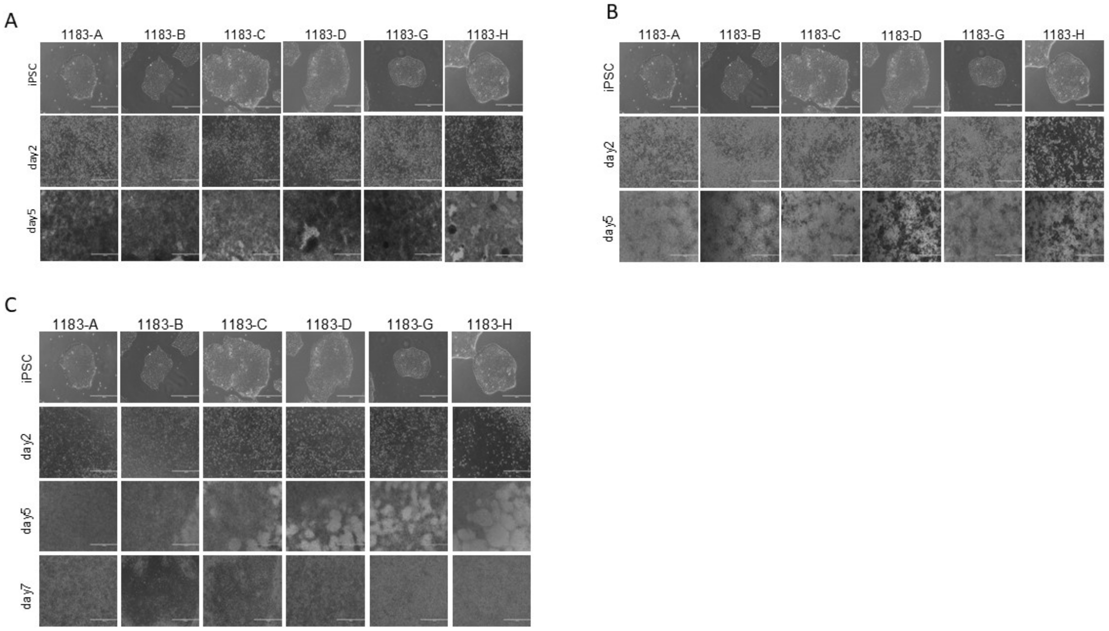

Figure S2. The differentiation of iPSC into the three germ layers (ectoderm, mesoderm, and endoderm) shown distinct morphological changes observed under a microscope. **A**. Mesoderm differentiation. **B**. Endoderm differentiation. **C**. Ectoderm differentiation.
